# The Treatment of Phthisis Night Sweats

**Published:** 1907-07

**Authors:** 


### The Treatment of Phthisis Night Sweats.

By Dr. Wilke, *Medico,* 1906, No. 52. The old remedies for nocturnal sweats in phthisis act only symptomatically. Since the sweats must be regarded as a biochemic reaction of the organism against the invasion of the blood stream by toxic substances, a causal remedy must be able to depoison the bacterial toxins and thus remove the entire symptom-complex,—the chills in the afternoon, the evening fever and the night perspiration. A priori, such an effect is to be anticipated from collargolum, as it has been shown that it acts katalytically on bacterial products.

Five phthisis patients in the second stage, with severe cough, mucopurulent expectoration and heavy night sweats, four of whom had moderate or high fever, received a tablespoonful of a 1% collargolum solution four times daily. After the use of ½ dram collargolum, fever and sweats disappeared and have not returned to this date, two months later. The importance of this result with reference to nutrition and strength is evident. When severe enteritis hinders absorption, inunction or intravenous injection of collargolum is preferable to its use by mouth. Collargolum does not act upon localised pus foci; therefore its use also has diagnostic value.

